# The Phenotypical and Functional Effect of PGE2 on Human Macrophages

**DOI:** 10.1002/eji.70090

**Published:** 2025-11-10

**Authors:** Maren Pfirrmann, Johanna Bödder, Rowan Wuestenenk, Jesse Tennebroek, Kirti K. lyer, Dennis Poel, Daniele V.F. Tauriello, Martijn Verdoes, I. Jolanda M. de Vries

**Affiliations:** ^1^ Department of Medical Biosciences Radboud University Medical Center Nijmegen The Netherlands; ^2^ Department of Medical Oncology Radboud University Medical Center Nijmegen The Netherlands; ^3^ Department of Medical Oncology, Erasmus MC Cancer Institute University Medical Center Rotterdam Nijmegen The Netherlands

**Keywords:** 3D tumor microenvironment, E‐prostanoid receptor, human macrophages, nanoparticles, prostaglandin E2

## Abstract

Prostaglandin E2 (PGE2) is one important immunosuppressive factor within the tumor microenvironment (TME). Signaling through E‐prostanoid receptor type 2 (EP2) and EP4, PGE2 promotes suppressive immune cell phenotypes and impairs antitumor immunity. Blocking PGE2 signaling with EP2 and EP4 antagonists is explored to counteract tumor‐induced immunosuppression. While tumor‐derived PGE2 is known to modulate human myeloid cell subsets, its specific effects on macrophages remain poorly defined. While murine models show PGE2 induces a protumorigenic macrophage phenotype, the role of PGE2‐EP2/4 signaling on human macrophages is unclear. This study evaluates the impact of PGE2 on human macrophage phenotype and function, and the effectiveness of targeting EP2 and EP4 with soluble and nanoparticle‐encapsulated antagonists. We show that PGE2 exposure during differentiation of monocytes to macrophages induces a distinct phenotype and affects macrophage functions. Tumor‐derived PGE2 predominantly signals through EP2; however, dual blockade of EP2 and EP4 more effectively counteracts PGE2‐induced changes. Notably, encapsulation of EP2/4 antagonists enhances the blockade of tumor‐derived PGE2 signaling on the macrophage phenotype and their ability to modulate T cell proliferation within patient‐derived tumor organoids. These findings underscore the influence of tumor‐derived PGE2 on human macrophages and support targeting the PGE2‐EP2/4 axis in cancer treatment.

## Graphical abstract

Prostaglandin E2 (PGE2) signaling through E‐prostanoid receptors (EP) 2 and 4 induces a distinct human macrophage phenotype and affects their functions. Targeting the PGE2‐EP2/4 axis in macrophages with soluble or nanoparticle‐encapsulated EP2/4 antagonists counteracts the PGE2‐induced effect in a 2D and 3D tumor microenvironment using patient‐derived tumor organoids.

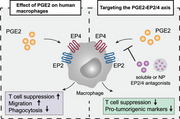

**Abbreviations**
CM: conditioned medium
COX: cyclooxygenases
DC: dendritic cell
aEP2 and aEP4: EP2 and EP4 antagonists
EP: E‐prostanoid receptor type
IFN‐γ: interferon‐gamma
IL‐10: interleukin‐10
LPS: lipopolysaccharide
M‐CSF: macrophage colony‐stimulating factor
BME: membrane extract type 2
MQ: Milli‐Q
MDMs: monocyte‐derived macrophages
MDSCs: myeloid‐derived suppressor cells
NPs: nanoparticles
BME: patient‐derived tumor organoids
PBS: phosphate‐buffered saline
PLGA: poly(lactic‐co‐glycolic) acid
PGE2: prostaglandin E2
TGF‐β: transforming growth factor‐beta
TME: tumor microenvironment
TAMs: tumor‐associated macrophages
wt%: weight percent

## 1 Introduction

The tumor microenvironment (TME) is a complex and dynamic system of various components, including tumor‐, stromal‐, immune cells, and soluble factors. Among these soluble factors, transforming growth factor‐beta (TGF‐β), interleukin‐10 (IL‐10), and prostaglandin E2 (PGE2) are commonly present in the TME [1, 2, 3]. These factors exert a suppressive effect on myeloid and lymphoid immune cells, leading to an overall immunosuppressive TME that facilitates cancer progression [1, 2, 4, 5, 6, 7]. One of the immunomodulatory factors, PGE2, is a lipid synthesized from arachidonic acid by cyclooxygenases (COX‐1 and COX‐2). Both COX‐2 and PGE2 are enriched in several cancer types [8, 9, 10, 11]. PGE2 mediates its effects by signaling through four E‐type prostanoid (EP) receptors (EP 1–4). EP2 and EP4 receptors are expressed on lymphocytes and myeloid cells, mediating the immunomodulatory effects of PGE2 [7, 12, 13, 14, 15, 16, 17]. In the myeloid cell population, it was found that tumor‐derived PGE2 signaling through EP2/4 induced a suppressive phenotype on dendritic cell (DC) subsets, resulting in the differentiation, activation, and tumor‐attraction of myeloid‐derived suppressor cells (MDSCs) [18, 19, 20, 21]. Moreover, PGE2 skews the polarization of human monocytes toward CD163^+^ M2 macrophages, which could be prevented by hindering PGE2 production with COX inhibitors [22]. In mice, PGE2 signaling through EP2 and predominantly EP4 has been identified as a factor that promotes the polarization of highly plastic macrophages toward a protumorigenic M2 phenotype [23, 24, 25]. Moreover, macrophage functions like migration or the secretion of chemokines and cytokines are affected in mice [26, 27, 28]. Bacterial infection together with PGE2 EP4 signaling increases CCL2‐dependent macrophage infiltration into gastric tumors in mice, whereas blocking PGE2 signaling with EP4 antagonists suppresses tumor development [27]. In human macrophages, PGE2‐EP4 signaling has been shown to suppress LPS‐induced chemokine production [29].

However, while the effects of tumor‐derived PGE2 on various human myeloid cell types have been well described, the impact of PGE2 signaling through EP2/4 on human macrophages and their function, particularly within the TME, remains to be further unraveled.

Macrophages stand out from the diverse immune cell population in the TME due to their high prevalence in solid tumors, their association with poor prognosis, and their notable plasticity [30, 31, 32]. They can be polarized via soluble factors to a wide range of reversible, functional states with proinflammatory M1 and anti‐inflammatory M2 macrophages representing two extremes in this dynamic continuum [33]. In vitro‐generated M1 macrophages, polarized with lipopolysaccharide (LPS) and interferon‐gamma (IFN‐γ), are defined by the expression of surface markers CD80 and HLA‐DR and display proinflammatory functions like T cell activation and secretion of inflammatory cytokines [34, 35]. PGE2 and cytokines like IL‐4, IL‐10, IL‐13, and TGF‐β promote M2 polarization [34, 36, 37, 38]. M2 macrophages are characterized by the expression of CD206 and CD163 and their execution of anti‐inflammatory functions as well as tissue remodeling and angiogenesis [34, 35]. The opposing functions of macrophages are crucial for a balanced immune response and particularly significant in the context of cancer. M1 macrophages can eliminate tumor cells, induce a robust proinflammatory immune response, and activate an adaptive T cell response [33]. In contrast, M2 macrophages promote a T helper 2 response, suppress T helper 1‐mediated inflammation through the secretion of IL‐10, and facilitate angiogenesis, thereby supporting tumor development [33, 39, 40, 41]. Within the immunosuppressive TME, macrophages are also known as tumor‐associated macrophages (TAMs), which often display a more M2‐like phenotype (39, 40, 41, 42]. Blocking and reversing the M2 polarization of TAMs into proinflammatory M1‐like macrophages is currently investigated as a promising approach in cancer immunotherapy [41].

Here, we propose targeting tumor‐derived PGE2 signaling in macrophages as a strategy to revert the protumorigenic M2 phenotype and enhance antitumor immunity. Although preventing PGE2 production with COX inhibitors has been shown to protect against cancer onset and progression, their use is limited due to cardiovascular and gastrointestinal adverse effects [43, 44, 45, 46]. A more specific alternative involves blocking EP2 and EP4 receptor signaling with specific antagonists, which has been described to avert immunosuppressive features across different myeloid cells like DCs and MDSCs [20, 21, 47, 48, 49]. Initial clinical trials with EP2 and EP4 antagonists are currently ongoing (NCT04344795, NCT05940571), and although the systemic administration of a soluble EP4 antagonist showed immunomodulation, it was accompanied by toxicity [50, 51].

Given the significance of PGE2‐EP2/4 signaling on myeloid cells in cancer and the high prevalence of macrophages within the TME, it is crucial to gain a deeper understanding of the importance of targeting the PGE2‐EP2/4 axis in human macrophages for the treatment of cancer.

In this study, we investigated the effect of PGE2 signaling on the phenotype and function of human macrophages. We observed that macrophages polarized with PGE2 showed functional, although not phenotypical, similarities to in vitro induced M2 macrophages. To resemble a physiologically relevant context, we used tumor‐derived PGE2 and novel 3D co‐cultures with patient‐derived tumor organoids (PDTOs). We showed that blocking EP2 and EP2/4 prevented PGE2‐induced suppression of macrophages, indicating that PGE2 mainly signals through EP2 to induce macrophage polarization. Macrophages within PDTO cultures demonstrated a suppressed phenotype and function, which could be reversed by blocking EP2/4. Additionally, the delivery of EP2/4 antagonists in nanoparticles [49, 52] showed a more robust prevention of the suppression of macrophages compared with the soluble application. Overall, these results demonstrate the important influence of tumor‐derived PGE2 on human macrophages and the therapeutic relevance of targeting the PGE2 EP2/4 axis for the treatment of cancer.

## 2 Results

### 2.1 PGE2 Influences Macrophage Phenotype During Differentiation, but Not During Polarization

**FIGURE 1:**
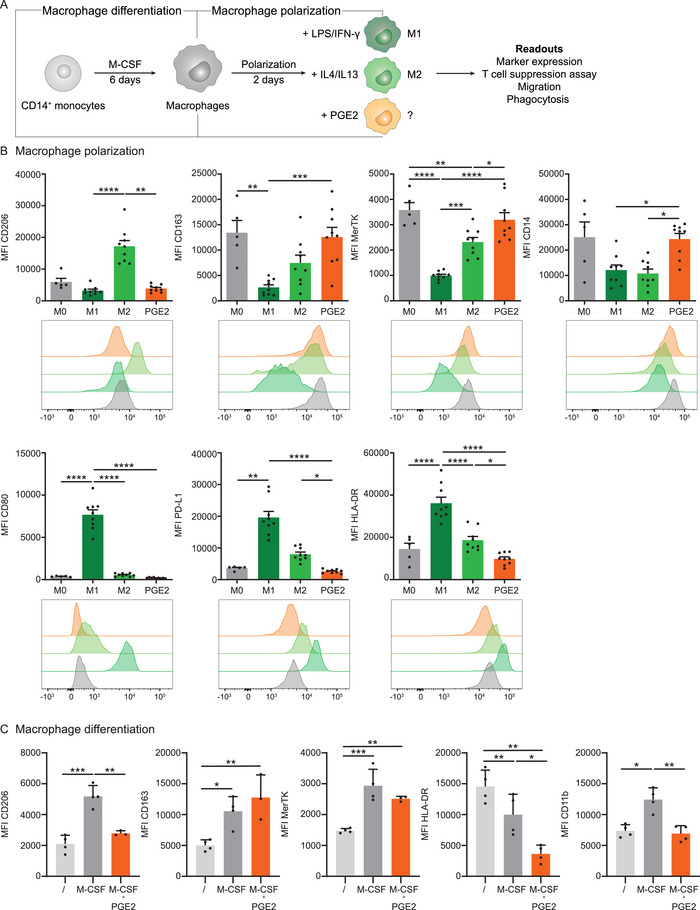
PGE2 impacts macrophage differentiation, but not macrophage polarization. (**A**) To determine the effect of PGE2 signaling on human macrophages, CD14^+^ monocytes were differentiated for 6 days with M‐CSF before polarizing them with LPS/IFN‐γ (M1), IL‐4/IL‐13 (M2), or PGE2. After 2 days of polarization, cells were analyzed for marker expression or used for functional assays such as T cell suppression, migration, or phagocytosis assays. (**B**) The marker expressions of polarized macrophages are depicted as bar graphs showing the mean fluorescent intensity (MFI) of CD206, CD163, MerTK, CD14, CD80, PD‐L1, and HLA‐DR. Bar graphs show the mean ± SEM, and each dot represents an individual donor (*n* ≥ 5) combined from two independent experiments. *p*‐values were calculated with one‐way ANOVA with Tukey multiple comparison correction or Kruskal–Wallis test with Dunn's multiple comparison correction. Additionally, histograms display the expression levels of each of those markers for one representative donor. (**C**) Monocytes nondifferentiated (/) or differentiated with M‐CSF, or M‐CSF + PGE2 for 6 days. MFI of CD206, CD163, MerTK, HLA‐DR, and CD11b is shown as bar graphs with the mean ± SEM, and each dot represents an individual donor (*n* = 4) from two independent experiments. *p*‐values were calculated using one‐way ANOVA with Tukey multiple comparison correction. *p < 0.05; **p < 0.01; ***p < 0.001; ****p < 0.0001.

To understand the impact of PGE2 on human macrophages, we started with the phenotypical consequence of PGE2 exposure on human monocyte‐derived macrophages (MDMs). Macrophage colony‐stimulating factor (M‐CSF) in vitro differentiated MDMs (M0 macrophages) were exposed to PGE2 (200 nM) for 2 days, after which we compared them to M1 (LPS + IFN‐γ) and M2 (IL‐4 + IL‐13) polarized macrophages, or to nontreated M0 macrophages (Figure 1A; Figure S1A). Notably, PGE2‐treated cells did not significantly differ from M0 cells when assessing the expression of typical M1 and M2 markers (Figure 1B). Like the nontreated M0 macrophages, PGE2‐treated macrophages showed a significantly lower CD206, PD‐L1, and HLA‐DR expression and a higher CD14 and MerTK expression compared with M2 macrophages (Figure 1B). Compared with M1 macrophages, CD163 and CD14 were significantly higher, and CD80, PD‐L1, and HLA‐DR were lower on PGE2‐exposed macrophages. Lower concentrations of PGE2 only slightly increased PD‐L1 expression, with no concentration‐dependent effect on other markers (Figure S1B).

Next, we studied the effect of PGE2 on the differentiation process of monocytes to macrophages, as monocyte differentiation is highly dependent on cues in the environment, such as PGE2 in the TME. Therefore, we incubated monocytes for 6 days in medium supplemented with either M‐CSF alone or M‐CSF with PGE2. The differentiation of CD14^+^ monocytes to macrophages with M‐CSF resulted in a significant upregulation of CD206, CD163, MerTK, and CD11b and a decrease in HLA‐DR expression compared with the nondifferentiated monocytes (Figure 1C). Thus, M‐CSF not only induces the upregulation of a typical macrophage marker, CD11b, but also results in a phenotypical profile resembling the M2 phenotype (Figure 1B). The combination of M‐CSF with PGE2 reduced the expression of CD206, CD11b, and HLA‐DR compared with M‐CSF treatment alone, while CD163 and MerTK expression were similar (Figure 1C; Figure S1C). Taken together, PGE2 induces changes in marker expression like CD206, HLA‐DR, and CD11b during differentiation of monocytes to macrophages, but does not seem to induce a distinct polarization state from M0 macrophages, based on the markers used in this panel.

### 2.2 PGE2‐Exposure of Macrophages Increases Suppression of T Cell Proliferation, Decreases Phagocytosis Capacity, and Increases Migration toward Tumor‐derived Signals

**FIGURE 2:**
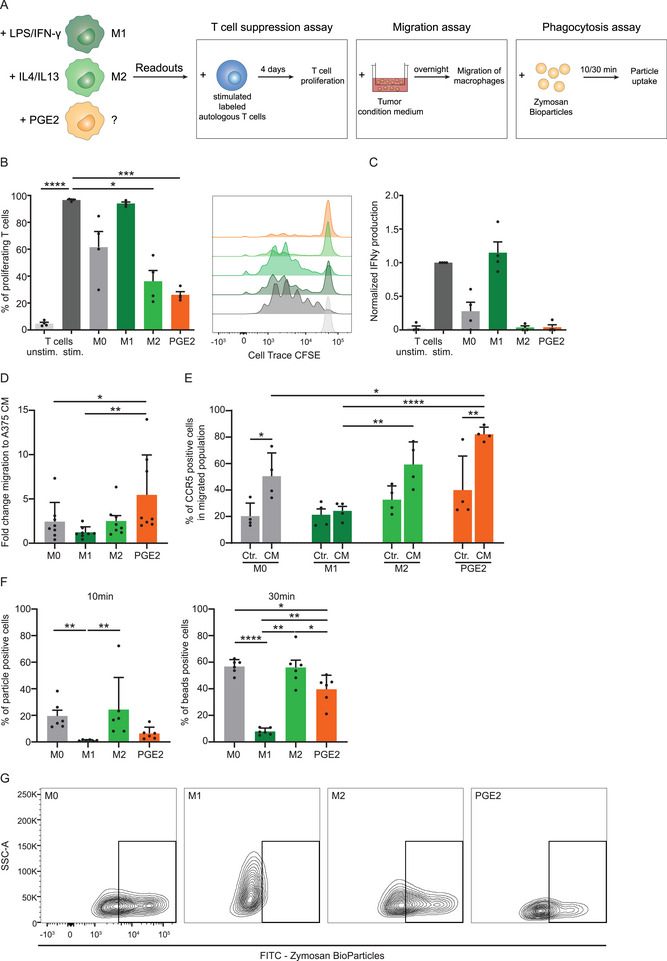
PGE2 signaling impacts human macrophage functions. (**A**) Graphical overview for various functional assays. To assess the suppression of T cell proliferation, M1‐, M2‐, and PGE2‐polarized macrophages were co‐cultured with stimulated, CFSE‐labeled autologous T cells for 4 days. M1‐, M2‐, and PGE2‐polarized macrophages were added to the upper chamber of a transwell migration assay. After overnight migration, the number of macrophages migrated to the lower chamber containing the tumor signal (A375 conditioned medium [CM]) was determined by flow cytometry. The phagocytic ability was analyzed by quantifying the uptake of Green Zymosan A BioParticles after 10 or 30 min incubation at 37°C or 4°C with flow cytometry. (**B**) Percentage of proliferating T cells co‐cultured with polarized macrophages compared with unstimulated or bead‐stimulated T cells alone. Bar graphs show the mean ± SEM with each data point representing one donor (*n* = 4) from one experiment. *p*‐values were calculated with one‐way ANOVA with Tukey multiple comparison correction. Additionally, histograms display the CFSE signaling for one representative donor. (**C**) IFN‐γ production detected via ELISA in the supernatant of the macrophage–T cell co‐culture normalized to the IFN‐γ production of stimulated T cells. Bar graphs display the mean ± SEM with each data point representing one donor (*n* = 4) from one experiment. *p*‐values were calculated on the raw data with one‐way ANOVA with Tukey multiple comparison correction. (**D**) Bar graph displays the mean ± SEM increased migration to A375 CM compared with passive migration (to control medium) for every polarized macrophage group. Each data point represents one donor (*n* = 8) combined from four independent experiments. *p*‐values were calculated with the Friedman test and Dunn's multiple comparison correction. (**E**) Percentage of CCR5^+^ macrophages in the migrated populations toward control medium (Ctr.) or A375 CM in M0, M1, M2, and PGE2 conditions. Bar graphs show the mean ± SEM, with each data point representing one donor (*n* = 4) combined from two independent experiments. *p*‐values were calculated with two‐way ANOVA with Tukey and Sidak multiple comparison correction. (**F**) Bar graphs show the percentage of Bioparticle positive polarized macrophages after 10 or 30 min of incubation at 37°C as a mean ± SEM, with each data point representing one donor (*n* = 6) combined from three independent experiments. *p*‐values were calculated with one‐way ANOVA with Tukey multiple comparison correction or Friedman test with Dunn's multiple comparison correction. (**G**) Dot blots display the uptake of Bioparticles in the FITC channel in polarized macrophages after 30 min incubation at 37°C. *p < 0.05; **p < 0.01; ***p < 0.001; ****p < 0.0001.

After establishing the phenotypical consequences of PGE2 exposure on macrophages, we aimed to get a better understanding of the functional impact of PGE2 on macrophages. PGE2 has been previously described to influence macrophage behavior in mice, but not in human [26, 27, 28]. Therefore, we assessed the effect of PGE2 on various macrophage functions, including suppression of T cell proliferation, migration, and phagocytic capacity. To study the effect of PGE2‐polarized macrophages on T cell proliferation, we co‐cultured polarized macrophages with autologous cell trace‐labeled (CFSE) pan T cells prestimulated with αCD3/αCD28 Dynabeads, after which we measured the reduction of CFSE fluorescence with flow cytometry as readout for T cell proliferation (Figure 2A). Despite the observed lack of impact of PGE2 on the expression of phenotypic markers, co‐culture with PGE2‐treated macrophages (200 nM) significantly reduced T cell proliferation, in contrast to M0 macrophages (Figure 2B). PGE2‐stimulated macrophages induced a similar suppression of T cell proliferation as M2 macrophages did. While the co‐culture of stimulated T cells with M1 macrophages produced similarly high IFN‐γ levels as the stimulated T cells alone, IFN‐γ secretion was reduced when T cells were cultured in the presence of M2 or PGE2‐polarized macrophages (Figure 2C). Further characterization of the T cell populations revealed that macrophage polarization did not affect the CD4/CD8 T cell ratio (Figure S2A). The inhibition of T cell proliferation is a strong indicator that PGE2 induces a suppressive state in macrophages.

Next, we investigated the migratory behavior of macrophages, as it has been reported that PGE2 influences the recruitment of other myeloid cells (e.g., MDSCs) toward the tumor. We performed a transwell migration assay with M1‐, M2‐, and PGE2‐polarized human macrophages toward tumor‐derived signals using A375 melanoma cell line‐derived conditioned medium (CM) (Figure 2A). PGE2‐polarized macrophages exhibited a significantly increased migratory capacity toward A375 CM compared with M0 and M1 macrophages (Figure 2D). Further, we determined the expression of CCR5 in macrophages, which was previously highlighted for its role in TAM accumulation in the TME [53]. Before migration, CCR5 was highly expressed on all macrophage subsets, with M1 macrophages exhibiting the lowest frequency of CCR5⁺ cells (Figure S2B). Following migration, the frequency of CCR5⁺ macrophages decreased under all conditions. Notably, a significantly higher proportion of CCR5+ macrophages migrated toward CM compared with control medium, except for M1 macrophages, whose percentage remained unchanged (Figure 2E). In PGE2‐polarized macrophages, the proportion of CCR5^+^ macrophages postmigration was comparable to premigration levels. This preserved CCR5⁺ percentage in PGE2‐polarized macrophages after migration implies that more CCR5⁺ cells actively migrated toward CM, suggesting responsiveness to tumor‐derived signals. Finally, we investigated the effect of PGE2 polarization on the phagocytic capacity of human macrophages with a Zymosan A BioParticle phagocytosis assay. The particle uptake by polarized macrophages was measured using flow cytometry after 10 and 30 min of incubation at 37°C (Figure 2A,G). Already after 10 min, M0 and M2 macrophages displayed a higher uptake compared with M1 macrophages (Figure 2F). Longer incubation with the particles increased particle uptake in most of the conditions. However, PGE2‐exposure significantly decreased the phagocytic activity compared with M0 macrophages, indicated by the low percentage of Zymosan A BioParticle‐positive cells. The MFI of the particle uptake followed the same trend, with reduced MFI in the M1 and PGE2‐polarized macrophages compared with M0 (Figure S2C). These results suggest that the addition of PGE2 decreases the phagocytic capacity of macrophages. Only minimal uptake is detected after 30 min of incubation at 4°C, suggesting active phagocytosis of the particles at 37°C (Figure S2D). Overall, PGE2 affects human macrophage functions by suppressing T cell proliferation, inhibiting their phagocytic abilities, and increasing the migration toward tumor‐derived signals.

### 2.3 EP2/EP4 Blockade Prevents Tumor‐Derived PGE2‐Induced Impact on Human Macrophages

**FIGURE 3:**
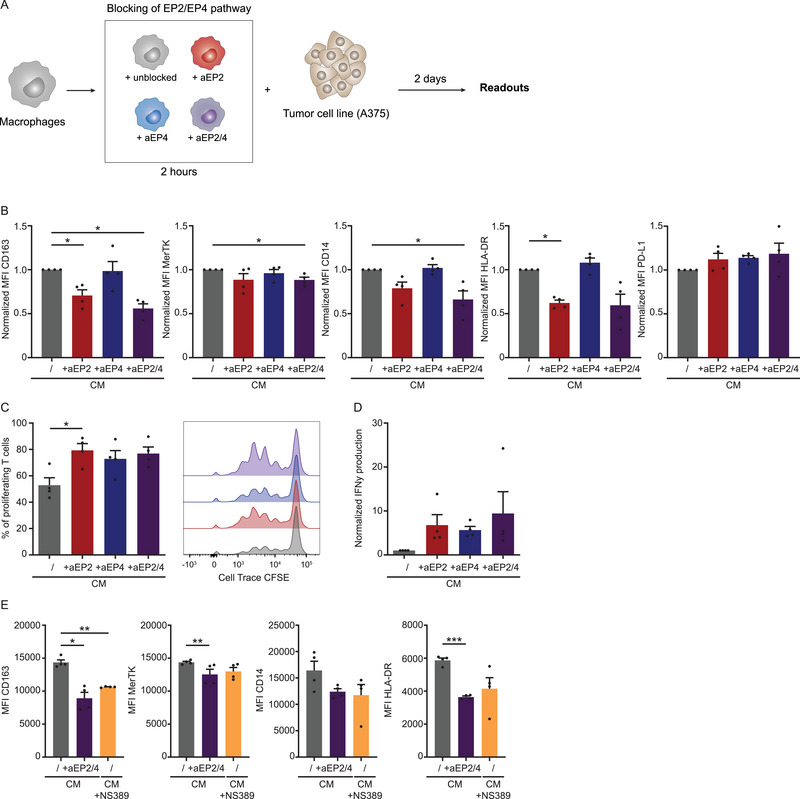
Prevention of tumor‐derived PGE2 signaling with EP2 and EP4 antagonists modulates macrophage phenotype and functions. (**A**) To determine the effect of blocking tumor‐derived PGE2 signaling on human macrophages, macrophages were treated with EP2 and EP4 antagonists (aEP2 and aEP4) alone, in combination, or unblocked (/) for 2 h. Subsequently, the cells were incubated with conditioned medium (CM) from A375 melanoma cells. After 2 days, the cells were analyzed for marker expression and their ability to influence T cell proliferation. (**B**) Bar graphs display the relative MFI of CD163, MerTK, CD14, HLA‐DR, and PD‐L1 normalized to the CM‐treated condition for every donor. Bar graphs show the mean ± SEM with each data point representing one donor (*n* = 4) combined from two independent experiments. *p*‐values were calculated on raw data with one‐way ANOVA with Tukey multiple comparison correction or Friedman test with Dunn's multiple comparison correction. (**C**) Percentage of proliferating T cells co‐cultured with treated macrophages. Bar graphs show the mean ± SEM, with each data point representing one donor (*n* = 4) from one experiment. *p*‐values were calculated with one‐way ANOVA with Tukey multiple comparison correction. Additionally, histograms display the CFSE signaling for one representative donor. (**D**) IFN‐γ production detected via ELISA in the supernatant of the treated macrophage T cell co‐culture normalized to the IFN‐γ production of CM‐stimulated macrophages. Bar graphs display the mean ± SEM, with each data point representing one donor (*n* = 4) from one experiment. *p*‐values were calculated with one‐way ANOVA with Tukey multiple comparison correction. (**E**) Expression of CD163, MerTK, CD14, and HLA‐DR on macrophages treated either with CM alone, CM with EP2/EP4 antagonists (aEP2 + aEP4), or CM from A375 cells pretreated with NS389 COX inhibitor to block PGE2 production. The bar graphs show the mean MFI ± SEM, with each data point representing one donor (*n* = 4) from one experiment. *p*‐values were calculated with one‐way ANOVA with Tukey multiple comparison correction or Friedman test with Dunn's multiple comparison correction. *p < 0.05; **p < 0.01; ***p < 0.001.

We next investigated the effect of tumor‐derived PGE2 on human macrophages to provide a more physiologically relevant stimulus to macrophages. Therefore, we exposed MDMs to CM from the melanoma cell line A375, known to produce PGE2 [49]. Additionally, we studied the inhibitory potential of EP2 and EP4 antagonists (AH6809/aEP2 and L‐161982/aEP4). We treated macrophages with aEP2 and aEP4 individually and in combination for 2 h before the cells were exposed to A375 CM. After 2 days, we analyzed the macrophage phenotype and the ability of macrophages to modulate T cell proliferation (Figure 3A). EP2 antagonists’ treatment resulted in the downregulation of CD163 and HLA‐DR expression, while aEP4 did not significantly modulate the CM‐induced phenotype (Figure 3B). The combination of both antagonists had a stronger effect on decreasing CD163, MerTK, and CD14 expression, as well as HLA‐DR to a lesser extent. Our findings indicate that blocking the EP2 receptor has a more pronounced effect, suggesting that PGE2 signals mostly through EP2 in macrophages. The impact on T cell proliferation followed a similar pattern, with aEP2 treatment of macrophages exposed to tumor‐CM reducing T cell suppression (Figure 3C). In addition, T cells co‐cultured with aEP2 and aEP4 and aEP2/4 combination‐treated macrophages showed a nonsignificant increase in IFN‐γ production (Figure 3D).

To show that the antagonists indeed block the PGE2‐mediated effects, we compared macrophages exposed to A375 CM with or without antagonists to macrophages exposed to A375 CM lacking PGE2. Therefore, we pretreated A375 cells with COX inhibitor NS398, which, as shown earlier, inhibits the PGE2 production in A375 cells and provides us with CM lacking PGE2 [49] (Figure 3A). The NS389‐treated CM led to a lower expression of CD163, MerTK, CD14, and HLA‐DR in macrophages, resulting in a phenotype similar to antagonist‐treated macrophages (Figure 3E). These results demonstrate that tumor‐derived PGE2 influences the human macrophage phenotype and that aEP2/4 treatment can prevent it. However, not all markers were equally affected by PGE2 signaling blockade, as CD206 and PD‐L1 expression were not significantly changed (Figure 3B; Figure S3A,B). Collectively, tumor‐derived PGE2 signaling modulates human macrophages primarily through EP2, although blocking both EP2 and EP4 could prevent PGE2‐induced changes more robustly.

### 2.4 EP Targeting Reduces Protumorigenic Marker Expression and Function in Macrophages Co‐Cultured with Patient‐Derived Tumor Organoids

**FIGURE 4:**
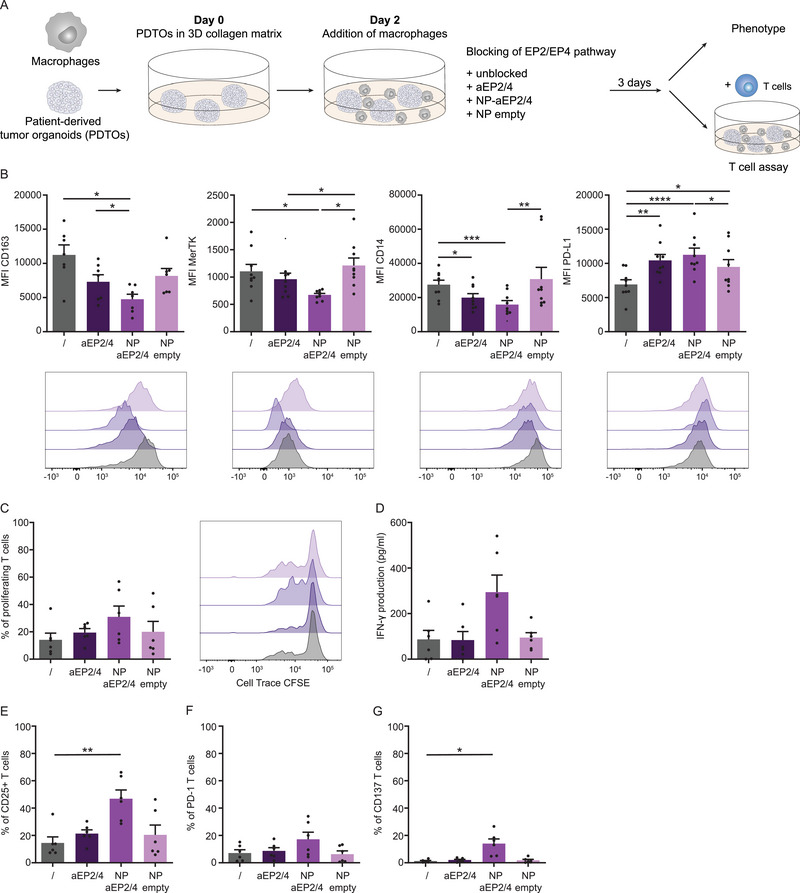
EP2/EP4 blocking modulates macrophage phenotype and functions in co‐culture with patient‐derived colorectal cancer organoids. (**A**) To investigate the effect of EP2/EP4 targeting of macrophages in a 3D model, patient‐derived tumor organoids (PDTOs) were cultured in a 3D collagen matrix for 2 days before adding macrophages from healthy donors (*n* = 6). The co‐culture was either left unblocked (/), treated with soluble aEP2/4, encapsulated aEP2/4 (NP‐aEP2/4), or with NP empty for 3 days before analyzing macrophage phenotype and the addition of T cells to check their ability to modulate T cell proliferation. (**B**) Bar graphs show the MFI of CD163, MerTK, CD14, and PD‐L1 on macrophages co‐cultured with PDTOs. Bar graphs show the mean ± SEM with each data point representing one donor (*n* ≥ 7) combined from four/five independent experiments. *p*‐values were calculated with one‐way ANOVA with Tukey multiple comparison correction or Friedman test with Dunn's multiple comparison correction. Additionally, histograms display the expression levels of each of those markers for one representative donor. (**C**) Percentage of proliferating T cells retrieved from PDTO macrophage co‐culture. Bar graphs show the mean ± SEM with each data point representing one donor (*n* = 6) combined from three independent experiments. *p*‐values were calculated with one‐way ANOVA with Tukey multiple comparison correction. Additionally, histograms display the CFSE signaling for one representative donor. (**D**) IFN‐γ production detected via ELISA in the supernatant of the macrophage, T cell, and PDTO co‐culture. Data are presented as bar graphs with mean ± SEM, and each data point represents one donor (*n* = 6) combined from three independent experiments. *p*‐values were calculated with one‐way ANOVA with Tukey multiple comparison correction. (E–G) Percentage of CD25, PD‐1, and CD137 positive T cells after retrieving from macrophage and PDTOs co‐culture. Bar graphs show the mean ± SEM with each data point representing one donor (*n* = 6) combined from three independent experiments. *p*‐values were calculated with one‐way ANOVA with Tukey multiple comparison correction or Friedman test with Dunn's multiple comparison correction. *p < 0.05; **p < 0.01; ***p < 0.001; ****p < 0.0001.

Next, we determined the effect of targeting tumor‐derived PGE2 signaling by blocking EP receptors in a 3D co‐culture model with macrophages and tumor organoids PDTO013, obtained from a colorectal cancer liver metastasis needle biopsy [54]. This model presents a more physiologically relevant context than a 2D plastic‐grown culture system [54, 55]. Furthermore, we detected low PGE2 production within the organoid co‐culture model (Figure S4A). In addition, we tested the encapsulation of EP2/4 antagonists in poly(lactic‐co‐glycolic) acid (PLGA) nanoparticles, as this approach may represent a potential drug delivery method toward phagocytic cells, such as macrophages. Previous studies displayed that nanoparticle‐encapsulated antagonists more effectively prevented PGE2‐induced suppression of the antitumor function of other myeloid cells [49, 52]. We added macrophages, together with soluble and encapsulated antagonists (NPs‐aEP2/4), as well as empty nanoparticles (NPs‐empty) as control, to the 3D collagen‐embedded PDTO culture [52]. After 3 days, we retrieved cells from the co‐culture, selected macrophages via CD45 staining, and assessed their phenotype (Figure 4A; Figure S4B).

The soluble aEP2/4 treatment resulted in significantly reduced CD14 expression and an increase in PD‐L1 expression (Figure 4B). The encapsulation of the antagonist enhanced this effect, decreasing not only CD14 but also CD163 and MerTK and significantly upregulating PD‐L1. Other markers like HLA‐DR, CD206, and CD80 displayed no changes in expression levels (Figure S4C). Notably, we observed that the encapsulated antagonists exerted their effect not only compared with the nontreated macrophages but also, for most markers, when compared with NP‐empty, indicating that this is an antagonist‐specific effect. However, we did detect reduced macrophage viability in both antagonist‐treated conditions, but not in the NP‐empty group (Figure S4D). Since macrophages are often recruited as monocytes to the TME, we also tested monocytes in the 3D co‐culture and treated them with soluble and encapsulated EP2/4 antagonists. Monocytes cultured for 3 days with PDTOs underwent similar phenotypical changes upon EP antagonist treatment as macrophages in co‐culture (Figure S4E). To test the functionality of the macrophages in the PDTO co‐culture, we assessed their ability to modulate T cell activation. Autologous, stimulated T cells, labeled with CFSE, were added to the co‐culture, and their proliferation and phenotype were measured after 3 days (Figure 4A; Figure S5A). We detected high proliferation of stimulated T cells in the 3D collagen culture, including in the presence of PDTOs. Whereas the addition to 3D PDTO cultures did not affect T cell proliferation, the addition to 3D M0 macrophage cultures suppressed it, with IFN‐γ production following a similar trend (Figure S5B,C). The co‐culture of macrophages and PDTOs resulted in reduced T cell proliferation in the same range as the macrophage single culture. Treatment of the macrophages with encapsulated antagonists for 3 days before the addition of stimulated T cells resulted in a slight increase in T cell proliferation, although not significantly. Neither the soluble antagonists nor the empty NPs showed a significant effect on T cell proliferation (Figure 4C). In addition, the IFN‐γ secretion displayed the same trend with increased IFN‐γ production after NP‐aEP2/4 treatment (Figure 4D). In line with this, NP‐aEP2/4 treatment showed higher expression of T cell activation markers like CD25, PD‐1, and CD137 compared with the other conditions (Figure 4E; Figure S5D). We conclude that the encapsulation of EP2/4 antagonists inside nanoparticles resulted in enhanced blockade of tumor‐derived PGE2 signaling on macrophage phenotype and their ability to modulate T cell proliferation within a 3D co‐culture model with PDTOs, indicating their potential for targeted therapeutic interventions in cancer immunotherapy.

## 3 Discussion

Tumor‐derived factors such as PGE2 are known to modulate immune cells toward a suppressive state in the TME [7, 47]. Despite the high abundance of PGE2 within different types of solid tumors and the high prevalence of macrophages within tumors, the impact of PGE2 on human macrophages has not been extensively studied. In this study, we demonstrate that PGE2‐EP2/4 signaling contributes to the induction of a suppressive phenotype and function of human macrophages in vitro. We show that the combined blockade of EP2/4, especially encapsulated in polymeric NPs, reduces protumorigenic marker expression and function of macrophages exposed to tumor‐derived PGE2 in 2D and a 3D co‐culture environment with PDTOs.

To get a deeper understanding of the PGE2 impact on human macrophages, we first investigated the phenotypical consequences of PGE2 in human monocyte‐derived macrophages. Interestingly, we did not observe any phenotypical differences between PGE2‐polarized and untreated M0 macrophages. However, in comparison to M1 and M2 macrophages, the PGE2‐treated cells displayed a lower expression of pro‐inflammatory M1 markers (CD80, HLA‐DR) and a higher expression of M2 markers (CD163, MerTK) [32, 34]. While we did not observe any alterations in macrophage phenotype, others showed that PGE2 signaling promotes M2 polarization based on cAMP activation [38, 56]. Although PGE2 did not induce phenotypic differences during macrophage polarization, our findings suggest that PGE2 plays a role during differentiation. As described in the literature, M‐CSF‐induced differentiation results in the upregulation of some M2 markers, shifting the macrophage phenotype toward a suppressive marker profile [57]. The addition of exogenous PGE2 to M‐CSF reduced CD206 and HLA‐DR expression and increased CD163 expression on macrophages. Furthermore, we measured decreased CD11b expression upon PGE2 treatment, indicating a hindered macrophage differentiation as previously reported [58]. In mice, PGE2 signaling hampered macrophage differentiation based on reduced CD11b expression, a process that could be reversed by blocking PGE2 signaling with EP antagonists or COX inhibitors [58]. While previous studies have investigated the effect of PGE2 on macrophages, the majority of findings are limited to murine studies. In our study, we solely focus on the impact PGE2 has on human macrophages. The differences between the findings in mice and our findings also highlight the importance of species differences.

Determining the effect of PGE2 on macrophages solely based on their phenotype is challenging due to interstudy variability in phenotypical markers and the range of polarization states of macrophages [59]. In human studies, macrophages and their polarization state in the TME are often based on single markers [59], while in mice, the characterization also relies on functionality and cytokine secretion [60]. It is established that the phenotype does not always correlate with the functionality of cells [59, 61]. Thus, to assess the effect of PGE2 on the polarization of human macrophages beyond the phenotype, we investigated crucial macrophage functions, including their ability to impact phagocytosis, migration, and T cell proliferation. Consistent with the PGE2‐induced suppressive effect on other myeloid cells, we showed that PGE2‐polarized macrophages inhibit T cell proliferation strongly than M2 macrophages [21]. Moreover, T cells co‐cultured with PGE2‐polarized macrophages produce no IFN‐γ, indicating a strong suppressive effect on T cells. This aligns with previous studies showing that PGE2‐treated macrophages impair T cell responses. T cell responses could be restored by inhibiting PGE2 signaling with EP antagonists [20, 60, 62].

Given that macrophages make up the majority of myeloid cells in TME, their migratory patterns are crucial. PGE2 has been shown to recruit myeloid cells, including macrophages, toward the tumor and alter their migratory behavior [60, 63]. Therefore, we studied the migration of polarized macrophages toward tumor signals using A375 CM, which contains PGE2 [49]. We observed increased migration of PGE2‐polarized macrophages compared with M0 or M1 macrophages. In mice, tumor‐induced macrophages with a skewed M2 phenotype also displayed increased migration, which could be prevented by blocking PGE2 signaling with EP antagonists [60]. In our data, we observed a higher percentage of CCR5^+^ migrated cells toward CM compared with control medium in the M0, PGE2, and, to a lesser extent, M2 conditions. Previous studies have shown that protumorigenic macrophages are recruited to tumors via the CCR5‐CCL5 signaling axis, and that blocking this pathway with CCR5 antagonists has improved clinical outcomes in cancer patients [53, 64, 65, 66]. While the proportion of CCR5+ cells among PGE2‐polarized macrophages after migration was comparable to premigration levels, their marked migration toward tumor‐derived signals suggests that CCR5 may contribute to this enhanced migratory response. However, further studies are required to confirm this hypothesis and elucidate the mechanisms underlying PGE2‐induced macrophage migration.

Phagocytosis is another important function of macrophages, which can be influenced by PGE2 signaling [60]. Our results show that PGE2‐exposed macrophages exhibited reduced phagocytosis compared with M2 and nontreated M0 macrophages. This finding is consistent with data from mice, demonstrating that PGE2 suppresses EP2‐mediated macrophage phagocytosis [60, 67]. In another study, PGE2 signaling through EP4 receptor suppressed phagocytosis in both M1 and M2 stimulated human macrophages [68]. Overall, these results demonstrate the suppressive effect of PGE2 on human macrophage functions and outline the potential of targeting the PGE2‐EP2/4 axis to prevent T cell exhaustion, reduce the accumulation of suppressive macrophages within the tumor, and restore phagocytic capacity. Our findings show the importance of evaluating multiple readouts to ensure a more accurate picture of the impact of PGE2 on macrophages.

To explore the potential of blocking tumor‐derived PGE2 signaling, we investigated the ability of EP2 and EP4 antagonists to reduce protumorigenic marker expression and function in macrophages. Using tumor‐derived PGE2 from A375 CM, the addition of aEP2 or aEP2/4 downregulated CD163, MerTK, and CD14 expression, which indicates a less suppressive macrophage phenotype [49]. Moreover, EP antagonists mitigated T cell suppression induced by CM‐treated macrophages. This shows the impact of EP2 and EP4 antagonists under a physiologically relevant condition using tumor‐derived PGE2 as stimulus.

The more pronounced effect observed when blocking EP2 suggests that PGE2‐EP2 signaling plays a crucial role in mediating the suppressive effects on macrophages. While some confirm the importance of PGE2‐EP2 signaling [67], others highlight EP4 signaling [20, 69]. In mice, EP4 inhibition has been shown to reduce tumor growth and repolarize macrophages toward an antitumor phenotype [20, 69]. Depending on the readout, such as T cell proliferation, the benefits of targeting EP4 become apparent. Consequently, we and others advocate for the dual blockade of EP2 and EP4 as a therapeutic advantage [21, 47, 48, 49].

We have shown that CM with COX inhibitor treatment, which resulted in PGE2 depletion as previously described, has a similar effect on the phenotype as EP2/4 antagonist treatment [49]. Previous studies demonstrated that reducing PGE2 levels in cancer patients with COX inhibitors can decrease cancer recurrence and cancer‐related deaths [44, 45]. However, the occurrence of adverse cardiovascular and gastrointestinal events shifted the focus toward using EP2 and EP4 antagonists, which are investigated in various clinical trials for treating solid cancers (NCT04344795, NCT05940571) [43, 46, 50, 51].

To account for the dynamic nature of the TME and the turnover of soluble factors like PGE2, we investigated the effect of soluble and encapsulated aEP2/4 in a more physiologically relevant, PGE2‐producing 3D patient‐derived tumor organoid co‐culture system. While 2D models are still commonly used, their inability to accurately mimic the TME makes them less favorable than 3D models. In our study, we first tested the phenotypical and functional impact of PGE2 in 2D before moving to our more complex and advanced 3D model to test the efficacy of encapsulated aEP2/4. By exploiting both human 2D and 3D models, we ensure the robustness of our findings. Our previous studies indicated that the encapsulation of aEP2/4 within polymeric NPs offers a new delivery approach toward phagocytic cells, potentially reducing side effects [49, 52]. Given that macrophages are known to take up NPs, this strategy could effectively counteract PGE2‐induced suppression [70]. Both soluble and encapsulated aEP2/4 downregulated CD163, MerTK, and CD14, while upregulating PD‐L1 expression in macrophages. Notably, NP‐aEP2/4 demonstrated superior efficacy compared with soluble antagonists, exhibiting a more pronounced downregulation of these suppressive markers. This downregulation can be attributed to the antagonists, as the empty control NPs did not show the same effect. However, macrophages that were treated with aEP2/4 or NP‐aEP2/4 displayed reduced viability as opposed to macrophages treated with empty NPs. Previously, PGE2 has been described as a survival signal for monocytes and macrophages, and the blockade of these signals with antagonists might increase cell death [71, 72]. Nevertheless, further investigations are required to explore the underlying cause and the effect that this might have on the phenotypical and functional state of the remaining macrophages. Furthermore, macrophages in mono‐ and co‐culture also demonstrated downregulated T cell proliferation. This suppression seems not exclusively induced by PGE2 signaling in macrophages because NP‐aEP2/4 was able to partially reduce T cell suppression. Of note, PGE2 is also recognized as a suppressor of T cells in the TME and has recently been shown to impair the effectiveness of tumor‐infiltrating lymphocytes [6, 17, 73], an effect that can be reversed with EP2/EP4 blocking. It would therefore be interesting to investigate the direct effect of PGE2 on T cells in a multicell organoid model and using NPs‐aEP2/4, functionalized to target T cells, to test their ability to prevent PGE2‐induced suppression. Furthermore, the increase of T cell activation markers (PD‐1, CD25, and CD137) showed that NP‐aEP2/4 can modulate T cell function in a co‐culture system [74, 75]. Since macrophages within the TME can inhibit T cell activity, leading to tumor progression [76], these results support the idea that NPs‐aEP2/4 exposure of macrophages in the TME results in a less suppressive phenotype, which could support antitumor immunity. These results, in addition to our previous findings, highlight the possibility of using NP‐aEP2/4 to target multiple phagocytic cells present in the TME.

Together, our findings suggest a pivotal role of PGE2‐EP2/4 signaling in inducing suppressive human macrophages within 2D and 3D tumor models. Notably, the combined blockade of EP2/4, via polymeric NPs‐aEP2/4, reduced the acquisition of this protumor state, highlighting the therapeutic value of targeting PGE2‐EP2/4 signaling axis to mitigate immunosuppression and boost the antitumor performance of macrophages in cancer.

## 4 Material and Methods

### 4.1 Cell Culture

Human melanoma A375 cells were cultured in Dulbecco's modified Eagle's medium (DMEM, ThermoFisher) with 10% fetal bovine serum (FBS) and 1% antibiotic‐antimycotic 7 (Gibco) at 5% CO_2_ and 37°C. To generate A375 conditioned medium (CM), 300,000 A375 cells per mL were plated in 2 mL in 6‐well plates. Moreover, to prepare PGE2‐depleted CM, A375 cells were treated with 10 µM NS‐398 (Cayman chemicals), a COX2 inhibitor. After 72 h, supernatants were collected and centrifuged for 5 min at 1500 rpm to exclude cell debris. Supernatants were frozen (−20°C) until further use.

Patient‐derived tumor organoids (PDTOs) were obtained from liver biopsies of patients with metastatic CRC included in the ORCHESTRA trial (NCT01792934). PDTO013 was developed and characterized as previously described [54, 77]. The PDTOs were cultured in 12 µL domes of Cultrex Reduced Growth Factor Basement Membrane Extract Type 2 (BME) (Bio‐Techne, 3533‐010‐02) in six‐well cell culture plates with 2 mL culture medium. As culture medium, we prepared +3 medium with Advanced DMEM/F12 (Gibco, 12634010) with 1% Penicillin‐streptomycin (Gibco, 15140122), 10 mM HEPES (Gibco, 15630056), and 1% GlutaMaxTM‐I (Gibco, 35050038). We further supplemented the +3 medium with the following: 5% (v/v) R‐spondin conditioned medium (provided by courtesy of the Kuo lab, Stanford University), 5% (v/v) Noggin‐CM (provided by courtesy of the Clevers lab, Hubrecht Institute), 1x B‐27 supplement without vit. A (Gibco, 12587010), 1x N2 supplement (Gibco, 17502048), 10 mM Nicotinamide (Sigma‐Aldrich, N0636), 0.2 mg/mL Normocin (InvivoGen, ant‐nr‐1), 1.25 mM N‐acetylcysteine (Sigma‐Aldrich, A9165), 10 nM human Gastrin‐I (Tocris, 3006), 50 ng/mL human recombinant EGF (Peprotech, AF‐100‐15‐1 mg), 2 µM Galunisertib/LY2157299 (DT), and 3 µM p38‐inhibitor/SB202190 (Selleckchem, S1077) as described before [54].

For the collection and passaging of PDTOs, the domes were mechanically disrupted with a pipette tip, collected in cold +3 medium, and centrifuged for 5 min at 1500 rpm. The remaining BME was removed from the pellet. Afterwards, PDTOs were mechanically broken down by pipetting up and down and centrifuged again for 5 min at 1500 rpm. For the passage of PDTOs, the pellet was resuspended in fresh 70% BME diluted with +3 medium, and the domes were added to a prewarmed six‐well plate. The plate was inverted and placed at 37°C for the BME to polymerize. Afterwards, 2 mL of +3 medium was added. To collect the PDTOs for co‐cultures, the retrieved pellet was trypsinized for 1 min at 37°C, vortexed, and mechanically broken down by pipetting. This process was repeated 2–3 times to generate single cells. The cells were counted and resuspended in +3 medium for further use in co‐culture experiments.

### 4.2 Cell Isolation

Peripheral blood mononuclear cells were isolated from buffy coats from healthy donors (Sanquin, Nijmegen, the Netherlands) via lymphoprep (VWR) density gradient centrifugation. CD14^+^ monocytes were isolated from Peripheral blood mononuclear cells with the MACS CD14^+^ isolation kit (130‐050‐201, Miltenyi Biotec) following the manufacturer's instructions. Afterwards, autologous pan T cells were isolated from the CD14‐negative fraction with the MACS Pan T cell isolation kit (130‐096‐535, Miltenyi Biotec) according to the manufacturer's protocols. The pan T cells were frozen in FBS with 10% Dimethyl sulfoxide (Merk) at −80°C until further use.

### 4.3 Differentiation and Polarization of Monocyte‐Derived Macrophages

To differentiate monocytes into monocyte‐derived macrophages (MDMs), CD14^+^ monocytes were cultured in MDM medium with macrophage colony‐stimulating factor (M‐CSF). As MDM medium, we prepared Roswell Park Memorial Institute 1640 medium (ThermoFisher) supplemented with 10% FBS, 1% stable glutamine (200 mM, CA‐STA‐B‐100 Westburg), and 1% antibiotic‐antimycotic. Isolated CD14^+^ cells were seeded at a concentration of 0.5 million per mL in six‐well plates with a final volume of 2 mL MDM medium with 50 ng/mL M‐CSF (300‐25‐100 Peprotech). The medium was refreshed after 3–4 days with M‐CSF, and the cells were differentiated for 6 days in total before various stimuli were added to polarize the MDMs. For co‐culture experiments, 1 million cells per mL were plated in T75 flasks with a final volume of 10 mL and differentiated for 7 days.

For the polarization, MDMs were either left for 2 days with 2 mL of MDM medium (M0), cultured with a final concentration of 100 ng/mL lipopolysaccharides (LPS) (Invivogen) and 20 ng/mL Interferon (IFN)‐y (Peprotech) (M1), or 20 ng/mL interleukin (IL)‐4 (Miltenyi Biotec) and 20 ng/mL IL‐13 (Miltenyi Biotec) (M2). Cells were stimulated with 0.2 µM PGE2, unless indicated differently. In the case of CM stimulation, 1 mL of A375 CM or CM treated with 20 µM COX inhibitor NS389 (Cayman chemicals) was added with 1 mL of MDM medium. To block the EP2/4 receptors, cells were treated with soluble EP2 antagonist (aEP2) (AH6809 Cayman Chemical) 45 µM, EP4 antagonist (aEP4) (L‐161982, Cayman Chemical) 2.5 µM, or a combination of a EP2/4 with the respective concentrations before CM polarization. After 2 h, 1 mL of A375 CM was added to a total of 2 mL medium.

Cells were stimulated for 48 h before harvesting. The cells were left for 30 min at 4°C with 2 mL of cold PBS + 2 mM ethylenediaminetetraacetic acid (EDTA) (Invitrogen, 15575‐020) before scraping off the MDMs and washing the wells with 1 mL of PBS + 2 mM EDTA. After retrieval, cells were further processed for flow cytometry staining or functional assays.

### 4.4 Monocyte Polarization

CD14^+^ monocytes were plated at a concentration of 0.5 million cells per mL in 2 mL of MDM medium in six‐well plates. The cells were either left untreated, differentiated with 50 ng/mL M‐CSF, or differentiated with 50 ng/mL M‐CSF with 0.2 µM PGE2. After 4 days, the MDM medium with stimuli was refreshed, and at day 6, the cells were harvested. In this experiment, floating cells in the supernatant were pooled with the attached population. Cells were prepared for staining, and marker expression was analyzed by flow cytometry.

### 4.5 Flow Cytometry Staining

Retrieved cells were washed with PBS in a 96‐well V‐bottom plate, and stained with viability dye eFluor780 (65‐0865‐14, ThermoFisher) 1:2000 in 25 µL PBS for 20 min at 4°C. Afterwards, the cells were washed with 100 µL PBA (PBS + 0.1% BSA + 0.01% NaH_3_) and stained depending on the experiment with different combinations of antibodies against CD163 BV421 (562643, BD Biosciences), CD206 FITC (551135, BD Biosciences), CD80 APC (305220, BioLegend), MERTK PE‐CY7 (367610, BioLegend), CD14 FITC (130‐113‐146, Miltenyi Biotec), PD‐L1 PE (557924, BD Biosciences), HLA‐DR BV510 (307646, BioLegend), and CD11b PE‐CY7 (A54822, Beckman) in 25 µL PBA for 20 min at 4°C. The cells were washed twice with PBA before measuring on a FACSVerse (BD Biosciences). The data were analyzed with the FlowJo v.10 software package (BD Biosciences).

### 4.6 T Cell Proliferation Assay

Previously isolated autologous pan T cells were thawed in MDM medium with DNAse I (Merck) and washed with PBS. In order to measure proliferation, the T cells were labeled with CellTrace CFSE (C34554, Invitrogen) using 1 µL per 10 million cells in 1 mL PBS + 1% FBS and incubated for 15 min at 37°C in the dark. Afterwards, 10 mL of FBS was added for 10 min and incubated at 37°C. The cells were washed with PBS, counted, and resuspended in 0.5 million cells per mL in X‐VIVO‐15 medium (Lonza) supplemented with 2% human serum (Sigma‐Aldrich). One million CFSE‐labeled T cells were stimulated with 10 µL αCD3/αCD28 human Dynabeads (Gibco) for 30 min at 37°C. Polarized MDMs were seeded with the CFSE‐labeled, stimulated T cells in a ratio of 1:1 with 50,000 cells each in a round‐bottom 96‐well plate in 200 µL X‐VIVO‐15 medium with 2% HS. Unstimulated and stimulated T cells without MDMs were seeded as controls.

After 4 days of incubation at 37°C, the supernatant was collected and stored at −20°C for subsequent IFN‐γ detection. The T cells were collected and washed with PBS. Live/dead staining was performed as described before, followed by a staining against CD8 APC (555369, BD Biosciences) and CD4 PE (555347, BD Biosciences) in 25 µL PBA for 20 min at 4°C. T cells were washed with PBA before being measured on a FACSVerse (BD Biosciences).

### 4.7 Migration Assay

To assess the migratory capacity of M1‐, M2‐, and PGE2‐polarized MDMs, a migration assay was performed using 96‐well transwell plates with a 5 µm pore size (CLS3388‐2EA, Corning Inc). The previously differentiated and polarized MDMs were harvested as described above, counted, and 36,000 cells were plated in the top well in 100 µL MDM medium. The lower chamber was filled either with 200 µL of MDM medium to determine passive migration or with 200 µL of A375 CM. After overnight migration at 37°C, the cells in the bottom chamber were detached with PBS + EDTA and washed with PBS. The migrated MDMs and the initially loaded starting population were stained with a live/dead marker (see above) and against CCR5 BV421 (306518, BioLegend) in 25 µL PBA for 20 min at 4°C. The cells were washed with PBA and measured for 45 s with the FACSLyric or FACS Verse (BD Biosciences). The percentage of migrated cells was calculated by dividing the number of live MDMs in the lower well by the number of initially loaded cells in the upper well. The active migration to CM was calculated as a fold change by dividing the migrated population to CM by the passively migrated population to MDM medium from the corresponding donor.

### 4.8 Phagocytosis Assay

The phagocytic capacity of stimulated MDMs was assessed with a phagocytosis assay using pHrodo Green zymosan A BioParticles (P35365, ThermoFisher). A total of 50,000 stimulated MDMs were detached and plated in a 96‐well U‐bottom plate with 40 µL zymosan A BioParticles in a final volume of 140 µL MDM medium. The zymosan A BioParticles were previously resuspended and sonicated according to the manufacturer's protocols. After 10 or 30 min of incubation at 37°C, or at 4°C as a control, the cells were collected and stained with a live/dead marker and against CD45 PerCP (B373771, BioLegend) as described above before measuring on a FACSVerse (BD Biosciences).

### 4.9 ELISA

IFN‐γ secretion from autologous T cells was measured in the cell‐free supernatant using the IFN‐γ ELISA kit (88‐7316‐88 Invitrogen) following the manufacturer's instructions. PGE2 level was determined in the cell‐free supernatant with the Prostaglandin E2 Human enzyme‐linked immunosorbent assay (ELISA) kit (EHPGE2 ThermoFisher) according to the manufacturer's protocol.

### 4.10 Nanoparticle Production and Characterization

The encapsulation of aEP2 and aEP4 in poly(lactic‐co‐glycolic) acid (PLGA) NPs was performed with the double emulsion solvent evaporation technique as described before [52]. In short, 3 weight percent (wt%) aEP2 and 2 wt% aEP4, dissolved in Dimethyl sulfoxide, were added to 100 mg PLGA (Evonik) in 3 mL dichloromethane (Merck). This organic phase was then added dropwise to 25 mL of 2.5% polyvinyl alcohol (Sigma Aldrich) and emulsified twice for 58 s at 20% amplitude. NPs without antagonists (empty NPs) were synthesized as controls. After solvent evaporation overnight at 4°C, the NPs were centrifuged at 11,000 rpm for 20 min at 4°C and washed three times with Milli‐Q (MQ) water before lyophilization. The average size and polydispersity index were determined by dynamic light scattering (Zetasizer, Nanotrace Flex), and the encapsulated antagonists were quantified as wt% and encapsulation efficiency measured using high‐performance liquid chromatography (Shimadzu, Japan) [52].

### 4.11 Co‐culture of MDMs, or Monocytes, With PDTOs

For the co‐culture of MDMs with PDTOs, we prepared 3D collagen layers in flat 96‐well plates as follows: 1.7 mg/mL rat tail collagen type 1 (IBIDI, 50203, Batch: 5NF210909FE and 5NF220930FE) with 5% (v/v) 10x MEM (Gibco, 11430‐030), 0.458% 0.5N NaOH, 11.85% MQ water, and 50% +3 medium. First, the neutralization mix with MEM, NaOH, and MQ water was prepared and kept on ice before the collagen and +3 medium were added. 50 µL of collagen mix was plated per well and polymerized for at least 1 h at 37°C. Afterwards, 30,000 dissociated PDTO single cells in 75 µL +3 medium were added on top of the collagen layer and incubated at 37°C for 2 days. Subsequently, 30,000 MDMs or monocytes in 50 µL MDM medium were added to the cultures. Additionally, 25 µL of MDM medium, medium with EP2/4a, or NPs were added to the wells. The soluble concentration of aEP2 was 120 µM, and for aEP4, 6 µM, while the concentration of nanoparticles (NPs) was adjusted to reach a final concentration of 120 µM aEP2, with aEP4 ranging from 3.1 to 4.3 µM depending on the NP batch. NPs without antagonists (NP empty) were used as a control in the same concentration. The NPs were dissolved in cold phosphate‐buffered saline (PBS) and added in 25 µL to the co‐cultures in a final volume of 150 µL. The co‐cultures were incubated at 37°C for 3 days.

To retrieve the cells from the collagen layers, we collected the gels and incubated them with 10% collagenase 1 (Sigma‐Aldrich, C0130) in PBS (20 U/L) for 15 min at 37°C and 300 rpm. The cell suspension was filtered with a 100 µm Cell Strainer (Corning, 431752), after which the cells were stained as described before.

To determine the effect of MDMs on T cell proliferation within the co‐cultures, we added autologous T cells to the wells. Therefore, we first replaced the medium of the co‐cultures with 100 µL of MDM and +3 medium in a 1:1 ratio. CFSE‐labeled T cells (see above) were resuspended in 1 million cells per mL X‐VIVO‐15 medium with 2% human serum, and 50,000 T cells in 50 µL were added on top of the co‐cultures and incubated for 3 days at 37°C. As controls, we seeded unstimulated and stimulated T cells without MDMs and PDTOs, and added stimulated T cells to MDM and PDTO mono‐cultures. The supernatant was collected and stored at −20°C for IFN‐γ detection. Cells were collected from the collagen layers and prepared for staining as described above. Live/dead staining was performed before staining against the following markers in 25 µL PBA for 20 min at 4°C: CD3 PE (555340, BD Biosciences), CD25 PE‐Cy7 (302612, BioLegend), PD‐1 BV421 (329920, BioLegend), and CD137 APC (550890, BD Biosciences). T cells were washed with PBA before being measured on a FACSVerse.

### 4.12 Statistical Analysis

Statistical analysis was conducted using GraphPad Prism 8 (Version 8.0.2. GraphPad Software, San Diego, CA, USA). Results are presented as mean ± SEM/SD in bar graphs with individual dots per donor. Normality was tested with the Shapiro–Wilk test. Significance between various conditions was calculated with a one‐way analysis of variance (ANOVA) followed by multiple comparison Tukey, Sidak correction, or the Friedman tests, followed by Dunn's multiple comparison test. The statistical significance is depicted as follows: *p < 0.05, **p < 0.01, ***p < 0.001, and ****p < 0.0001.

## Author Contributions

Maren Pfirrmann and Johanna Bödder designed the research. Maren Pfirrmann, Johanna Bödder, Rowan Wuestenenk, and Jesse Tennebroek performed the experiments and analyzed the data. Daniele V.F. Tauriello, Kirti K. Lyer, and Dennis Poel provided patient‐derived tumor organoid material. Maren Pfirrmann and Johanna Bödder wrote the manuscript. Martijn Verdoes and I. Jolanda M. de Vries supervised and assisted with data interpretation. Martijn Verdoes and I. Jolanda M. de Vries acquired funding. All the authors reviewed and approved the submission of the manuscript.

## Funding Information

This work was supported by a Radboudumc PhD grant and Health∼Holland grant DC4Balance (LSHM18056‐SGF).

## Conflicts of Interest

The authors declare no conflicts of interest.

## Ethics Statement

Human monocytes and T cells were isolated from buffy coats and apheresis products (Sanquin, Amsterdam, the Netherlands) from healthy donors after written consent and according to institutional guidelines.

## Supporting information




**Supporting File 1**: eji70090‐sup‐0001‐SuppMat.pdf

## Data Availability

The data supporting the findings of this study are available from the corresponding author upon reasonable request.
