# Supplementary material for: The Phenotypical and Functional Effect of PGE2 on Human Macrophages
Source: Eur J Immunol. 2025 Nov 10;55(11):e70090. doi: 10.1002/eji.70090 (PMC12599275; doi:10.1002/eji.70090)
Supplement: Supplementary file 1 — Supporting File 1: eji70090‐sup‐0001‐SuppMat.pdf [file EJI-55-e70090-s001.pdf]

Supplementary Information

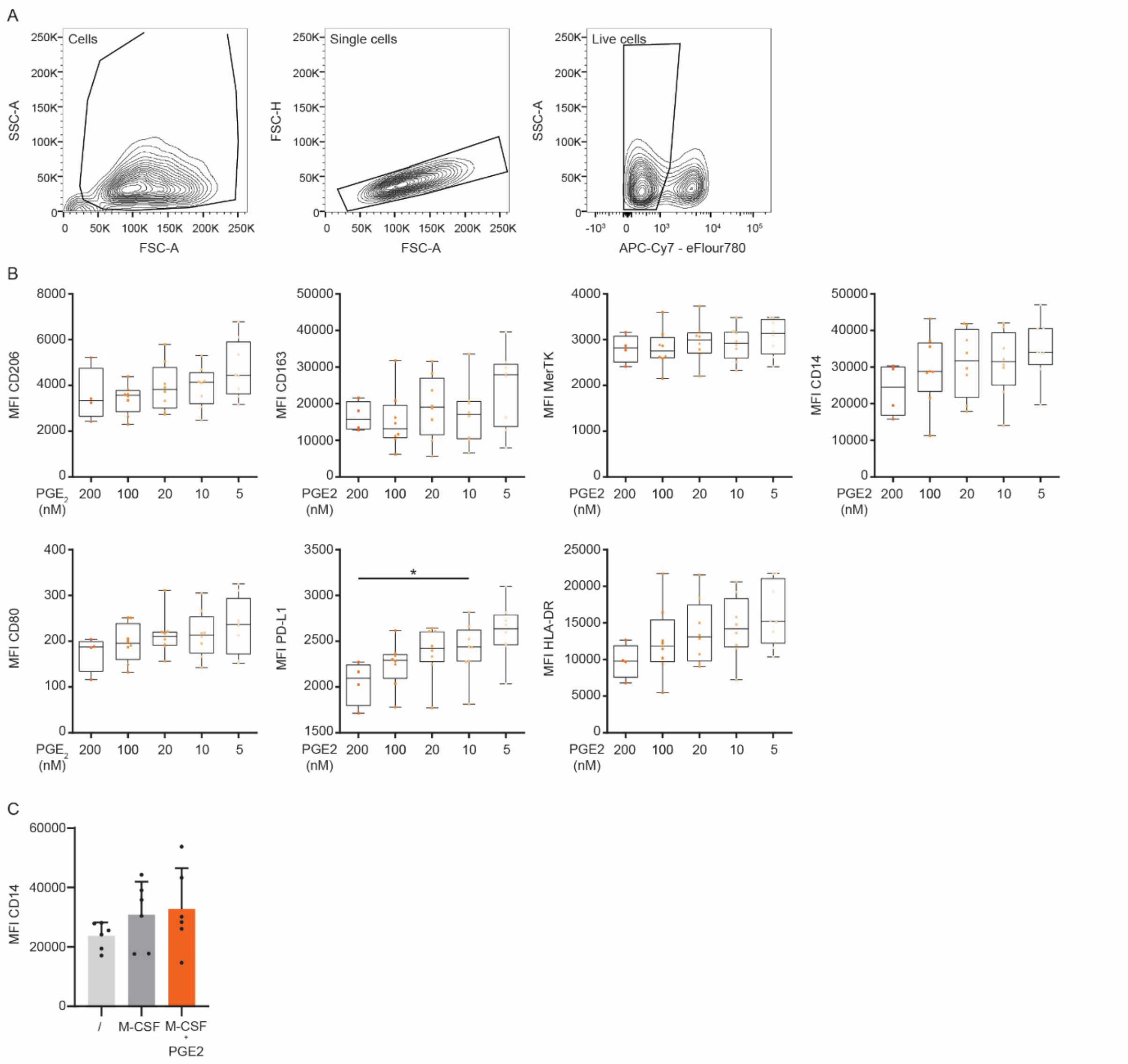

Figure 1. (**A**) Gating strategy to assess the polarized macrophage phenotype. The first gate is set based on FSC-A / SSC-A followed by FSC-A / FSC-H to identify the single cell population. Next, the living cells are determined as APC-Cy7 (eFluor780) negative before gating on the separate markers. (**B**) Graphs depict the mean fluorescent intensity (MFI) of CD206, CD163, MerTK, CD14, CD80, PD-L1, and HLA-DR in macrophages polarized for 2 days with indicated PGE2 concentrations. Box plots show the mean ± SEM and each dot represents an individual donor (n=8) combined from two independent experiments. P values were calculated with one-way ANOVA with Tukey multiple comparison correction. (**C**) MFI of CD14, on monocytes polarized with M-CSF or M-CSF + PGE2 for 6 days. Bar graphs show the mean ± SEM and each dot represents an individual donor (n=4) from two independent experiments. P values were calculated with one-way ANOVA with Tukey multiple comparison correction or Friedman test with Dunn's multiple comparison correction. P* < 0.05.

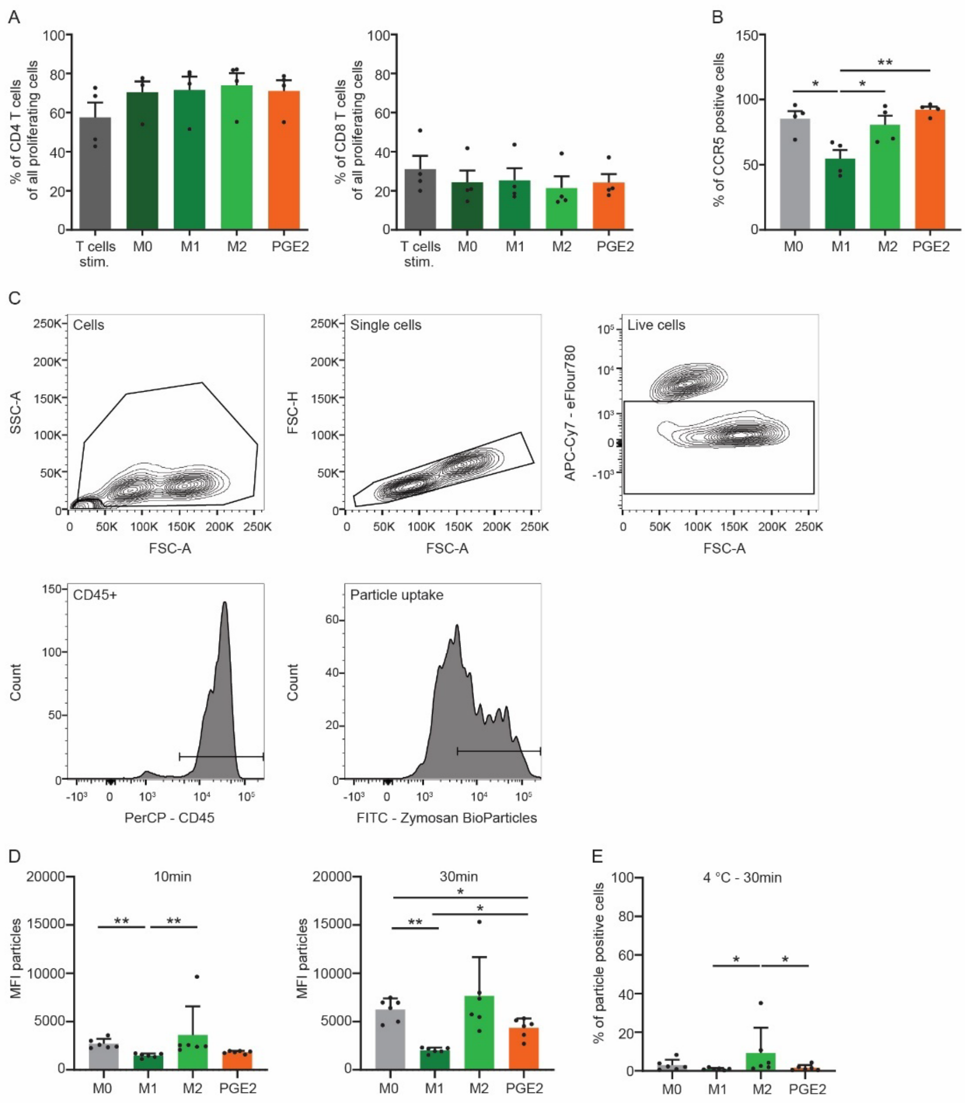

Supplementary Figure 2. (**A**) Percentage of CD4 and CD8 proliferating T cells co-cultured with polarized macrophages, compared to beads-stimulated T cells alone. Bar graphs show the mean ± SEM with each data point representing one donor (n=4) from one experiment. P values were calculated with Friedman test with Dunn's multiple comparison correction. (**B**) Percentage of $CCR5^+$ macrophages before migration assay within the different conditions. Bar graphs show the mean ± SEM with each data point representing one donor (n=4) combined from two independent experiments. P values were calculated with one-way ANOVA with Tukey multiple comparison correction. (**C**) Gating strategy to determine uptake of Zymosan particles in macrophages. From the FSC-A / SSC-A gate, the single cells were identified by FSC-A / FSC-H before determining the living cells as APC-Cy7 (eFluor780) negative. The particle uptake was detected within the CD45-positive cell population. (**D**) Bar graphs show the percentage of Bioparticle-positive polarized macrophages after 30 minutes of incubation at 4 °C as mean ± SEM with each data point representing one donor (n=6) combined from three independent experiments. P values were calculated with Friedman test with Dunn's multiple comparison correction. (**E**) Bar graphs depict the MFI of particle-positive polarized macrophages after 10- or 30-minute incubation at 37 °C as mean ± SEM with each data point representing one donor (n=6) combined from three independent experiments. P values were calculated with one-way ANOVA with Tukey multiple comparison correction or Friedman test with Dunn's multiple comparison correction. $P^* < 0.05$, and $^{**}p < 0.01$.

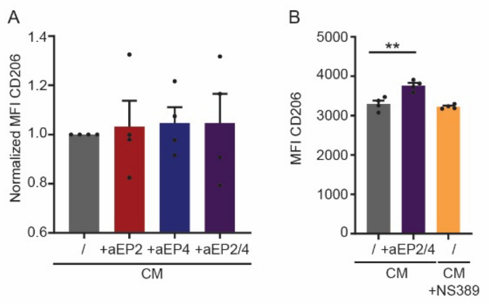

Supplementary Figure 3. (**A**) The relative MFI of CD206 after CM or CM with EP2 antagonists (aEP2), aEP4, or the combination (aEP2/4) treatment. Bar graphs show the MFI normalized to CM treated condition for every donor as mean ± SEM with each data point representing one donor (n=4) combined from two independent experiments. P values were calculated on raw data with one-way ANOVA with Tukey multiple comparison correction. (**B**) Expression of CD206 on macrophages treated either with CM alone, CM with aEP2/4 or CM from NS389-treated A375 cells. The bar graphs show the mean MFI ± SEM with each data point representing one donor (n=4) from one experiment. P values were calculated with one-way ANOVA with Tukey multiple comparison correction. **P < 0.01.

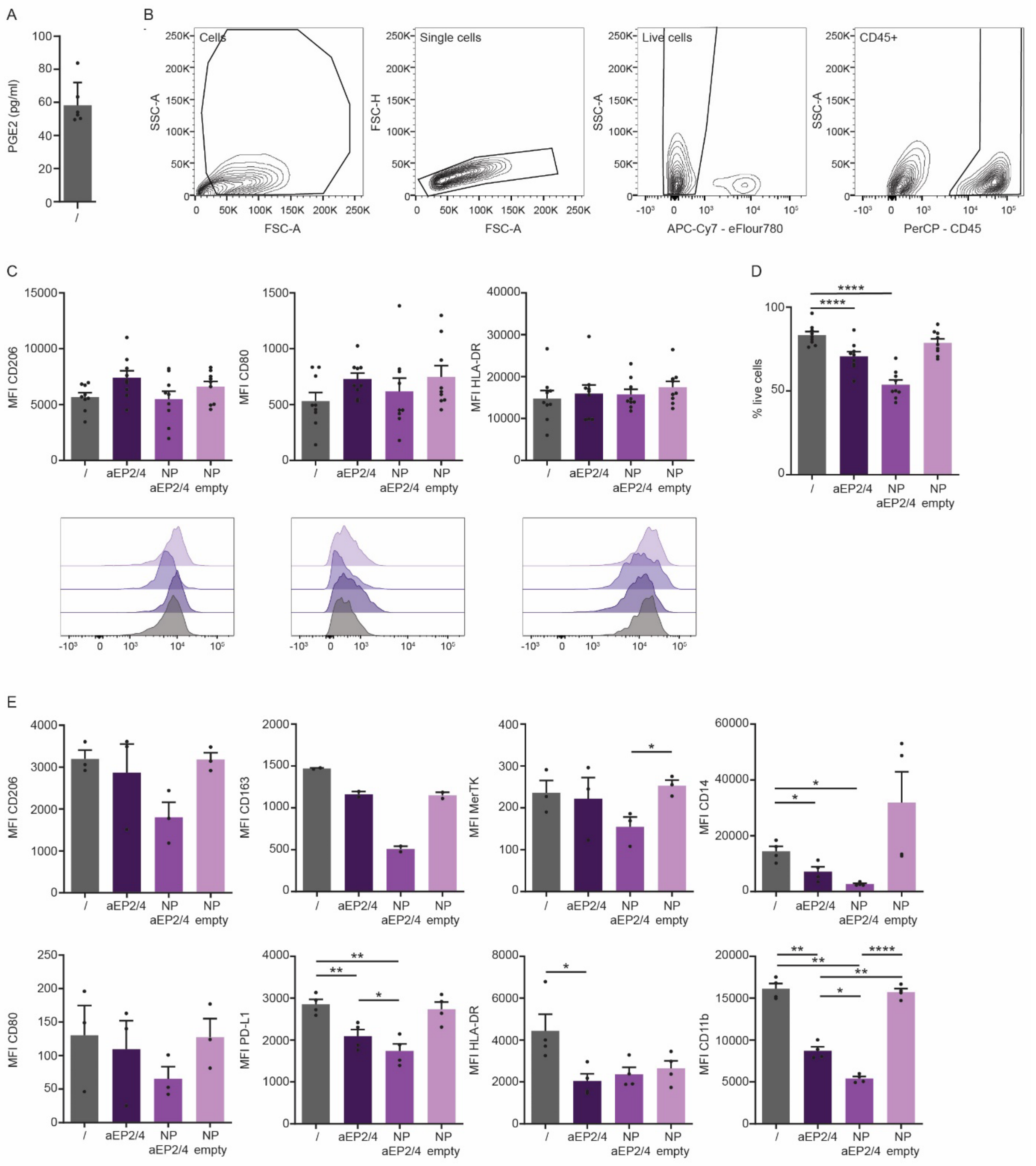

Supplementary Figure 4. (**A**) PGE2 secretion in co-culture measured with ELISA. Bar graphs show the mean ± SEM and each dot represents an individual donor (n=6) from three independent experiments. (**B**) Gating strategy to assess the phenotype of macrophages retrieved from co-culture with patient-derived tumor organoids (PDTOs). The first gate is set based on FSC-A / SSC-A followed by FSC-A / FSC-H to identify the single cell population. The living cells are determined as APC-Cy7 (eFluor780) negative before gating on CD45-positive macrophages and the separate markers from this population. (**C**) Expression levels of CD206, CD80, and HLA-DR on macrophages co-cultured with PDTOs untreated (/), treated with soluble antagonists (aEP2/4), encapsulated EP2/4 antagonists (NP-aEP2/4), or empty NPs. Bar graphs show mean MFI ± SEM with each data point representing one donor (n=9) combined from five independent experiments. P values were calculated with one-way ANOVA with Tukey multiple comparison correction or Friedman test with Dunn's multiple comparison correction. Additionally, histograms display the expression levels of each of those markers for one representative donor. (**D**) Percentage of live macrophages after co-culturing with PDTOs and different antagonist treatments. Bar graphs show mean ± SEM with each data point representing one donor (n=9) combined from five independent experiments. P values were calculated with one-way ANOVA with Tukey multiple comparison

correction. (**E**) Expression of different markers expressed on $CD14^+$ monocytes co-cultured for 2 days with PDTOs and different antagonist treatments. The bar graphs show the expression of CD206, CD163, MerTK, CD14, CD80, PD-L1, HLA-DR, and CD11b as mean MFI ± SEM with each data point representing one donor ($n \geq 2$) combined from one/two independent experiments. P values were calculated with one-way ANOVA with Tukey multiple comparison correction. P* < 0.05, **p < 0.01, and ****p < 0.0001.

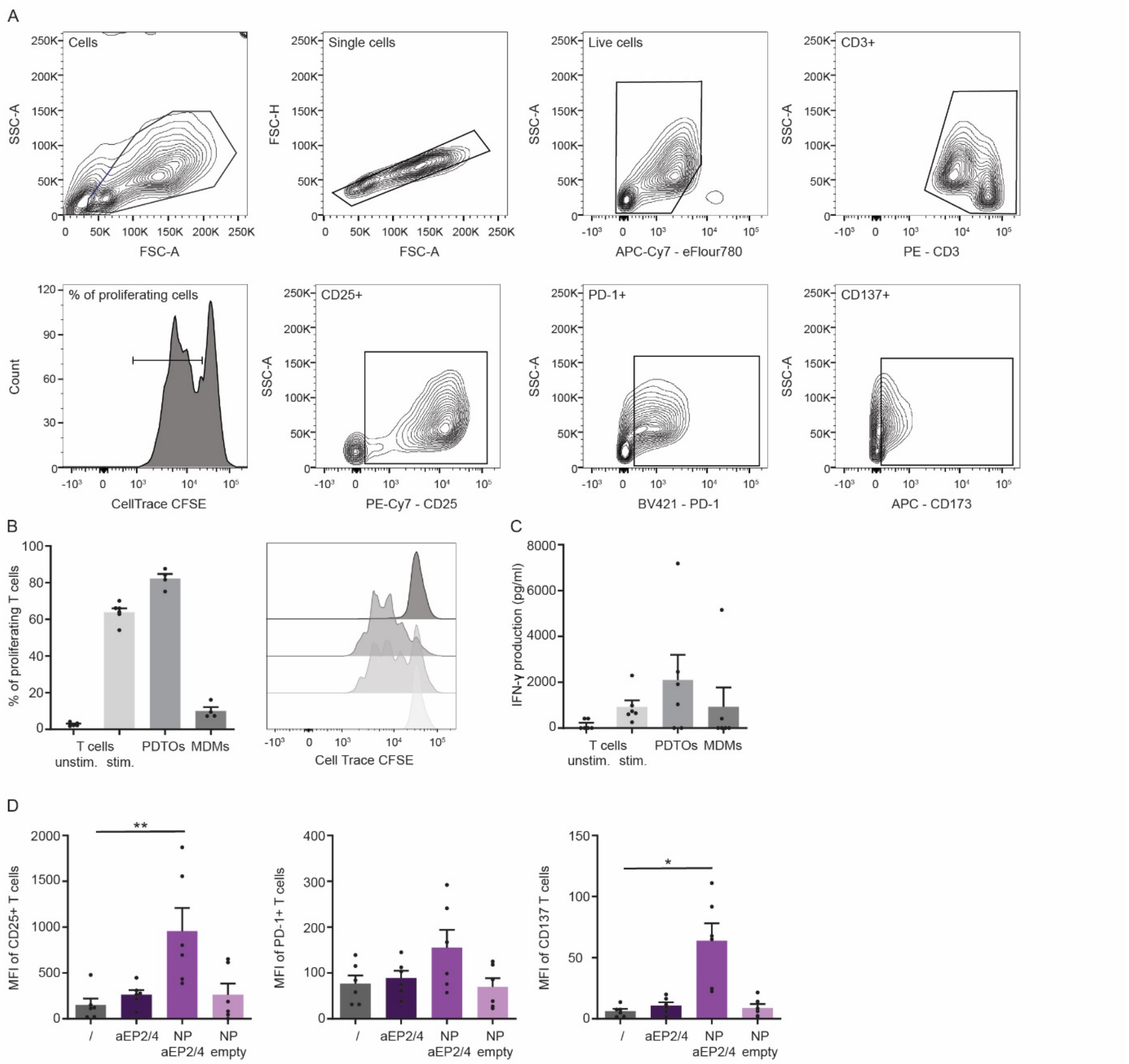

Supplementary Figure 5. (**A**) Gating strategy to analyze T cell activation after co-culture with macrophages and patient-derived tumor organoids (PDTOs) and different antagonist treatments. Cells were gated based on FSC-A / SSC-A followed by FSC-A / FSC-H to identify the single cell population. The living cells are determined as APC-Cy7 (eFluor780) negative before identifying T cells as CD3-positive cells. From the CD3 positive cells, T cell proliferation was gated via the loss of CFSE signaling and the expression of T cell activation markers CD25, PD-1, and CD137. (**B**) Percentage of proliferating T cells retrieved from PDTO macrophage co-culture of unstimulated (T cells unstim), beads stimulated T cells (T cells stim), or stimulated T cells co-cultured with PDTOs alone (PDTOs) or macrophages alone (MDMs). Bar graphs show the mean ± SEM with each data point representing one donor (n≥4) combined from two independent experiments. Additionally, histograms display the CFSE signaling for one representative donor. (**C**) IFN-γ production detected via ELISA in the supernatant. Data is presented as bar graphs with mean ± SEM and each data point representing one donor (n=6) combined from three independent experiments. P values were calculated with one-way ANOVA with Tukey multiple comparison correction. (**D**) Expression of CD25, PD-1, and CD137 on T cells after retrieving them from macrophage and PDTOs co-culture left untreated (/) or treated with soluble antagonists (aEP2/4), encapsulated antagonists (NP-aEP2/4), or empty NPs. Bar graphs show the mean ± SEM with each data point representing one donor (n=6) combined from three independent experiments. P values were calculated with one-way ANOVA with Tukey multiple comparison correction or Friedman test with Dunn's multiple comparison correction. P* < 0.05, and **p < 0.01.
